# Accuracy of ventilator-associated events for the diagnosis of ventilator-associated lower respiratory tract infections

**DOI:** 10.1186/s13613-020-0624-6

**Published:** 2020-01-13

**Authors:** Olivier Pouly, Sylvain Lecailtel, Sophie Six, Sébastien Préau, Frédéric Wallet, Saad Nseir, Anahita Rouzé

**Affiliations:** 1 Critical Care Center, CHU Lille, 59000 Lille, France; 2 Medicine Faculty, Lille University, 59000 Lille, France; 3 Intensive Care Unit, CH de Boulogne, Boulogne, France; 4 Centre de Biologie et de Pathologie, CHU Lille, 59000 Lille, France

**Keywords:** Ventilator-associated events, Ventilator-associated tracheobronchitis, Ventilator-associated pneumonia

## Abstract

**Background:**

The aim of this study was to investigate the concordance between ventilator-associated events (VAE) and ventilator-associated lower respiratory tract infections (VA-LRTI), and their impact on outcome.

**Methods:**

This retrospective study was performed in five 10-bed ICUs of a teaching hospital, during a 2-year period. Ventilator-associated lower respiratory tract infections (VA-LRTI), including ventilator-associated tracheobronchitis (VAT) and ventilator-associated pneumonia (VAP) were prospectively diagnosed. The agreement between VAE, VAT and VAP was assessed by k statistics.

**Results:**

A total of 1059 patients (15,029 ventilator-days) were included. 268 VAP (17.8 per 1000 ventilator-days), 127 VAT (8.5 per 1000 ventilator-days) and 262 VAE (17.4 per 1000 ventilator-days) were diagnosed. There was no agreement between VAT and VAE, and the agreement was poor between VAP and VAE (*k* = 0.12, 95% CI 0.03–0.20). VAE and VA-LRTI were associated with significantly longer duration of mechanical ventilation, ICU and hospital length of stay. VAP, VAT and VAE were not significantly associated with mortality in multivariate analysis.

**Conclusions:**

The agreement was poor between VAE and VAP. No agreement was found between VAE and VAT. VAE episodes were significantly associated with longer duration of mechanical ventilation and length of stay, but not with ICU mortality.

## Background

In spite of increased use of non-invasive mechanical ventilation, and high-flow nasal oxygen in the intensive care unit (ICU), invasive mechanical ventilation is still used in a large proportion of critically ill patients [1]. Ventilator-associated lower respiratory tract infections (VA-LRTI), including ventilator-associated pneumonia (VAP), and ventilator-associated tracheobronchitis (VAT) are the most common complications in patients receiving mechanical ventilation. These infections are associated with increased duration of mechanical ventilation, length of hospital stay, and cost [2, 3].

The diagnosis of these infections is based on chest X-ray, which is not specific in detecting new infiltrates in critically ill patients. Therefore, the CDC recommended using a new definition for ventilator-associated events (VAE), including infectious and other conditions. This definition includes only objective criteria and is perfectly reproducible [4]. However, recent studies and meta-analysis reported poor agreement between VAE, including ventilator-associated conditions (VAC), infection-related ventilator-associated complications (IVAC), or probable VAP (pVAP) [5–7]. Few studies evaluated the agreement between VAE and VA-LRTI, including VAP and VAT. Although VAP and VAT are both associated with increased duration of mechanical ventilation and length of ICU stay, only VAP is associated with increased mortality rates [8]. Thus, it is probably important to distinguish VAP from VAT. In addition, the recent ATS/IDSA guidelines on VAP recommended not treating VAT patients with antimicrobial, based on the low quality of the available evidence [9]. Therefore, we conducted this retrospective analysis of prospectively collected data to determine the agreement between VAE and VA-LRTI, including VAP and VAT. We also aimed to determine the impact of VAE on outcomes, including duration of mechanical ventilation, length of ICU and hospital stay, and mortality.

## Patients and methods

### Study design

This study was conducted in five 10-bed ICUs in Lille University Hospital, during a 2-year period (from January 1st, 2016 through December 31st, 2017). The IRB of the Lille University Hospital approved the study and waived informed consent. In accordance with the French law, and because of the retrospective observational design, written informed consent was not required. All patients hospitalized in one of the 5 ICUs and receiving invasive mechanical ventilation for at least 5 days were eligible for this study. Patients who received mechanical ventilation for < 5 days, and those who received mechanical ventilation for > 24 h before ICU admission were excluded.

### Definitions

VA-LRTI included VAP and VAT. VAP was defined as pneumonia diagnosed after 48 h of intubation and mechanical ventilation. The diagnostic criteria for VAP included a new infiltrate on chest X-ray associated with at least two of the following: body temperature ≥ 38.5 °C or < 36 °C; leukocyte count ≥ 10 × 10^9^/L or < 1.5 × 10^9^/L; and purulent tracheal aspirate or sputum. In addition, a microbiological confirmation was required for all patients (positive endotracheal aspirate culture ≥ 10^5^ colony-forming units (cfu)/mL or positive bronchoalveolar lavage culture ≥ 10^4^ cfu/mL) [9]. VAT was defined using the same criteria as for VAP, except the presence of new or progressive pulmonary infiltrate. VAE were diagnosed according to CDC definition (Additional file 1: Figure S1). VAE diagnosis was considered concordant with that of VAT or VAP, when these infections occurred within 2 days before or after the alteration of PEEP or FiO2 (Additional file 1: Figure S2).

### VAP prevention and treatment

A VAP prevention strategy was routinely used during the study period. The ventilator circuit was not changed routinely. Sedation and weaning were based on a written protocol. A minimal positive end expiratory pressure of 5 cm H_2_O was used in all patients. Oral cavity was cleaned with chlorhexidine thrice daily. Cuff pressure was measured and adjusted (25 cm H_2_O) by nurses thrice a day. Tracheal suctioning was routinely performed by nurses, using an open tracheal suction system. Patients remained in semi-recumbent position, and received enteral nutrition based on a written protocol.

Antibiotic treatment for patients with suspected VAP was based on ATS/IDSA guidelines [9]. Antibiotic treatment for other infections was based on written local guidelines adapted from international and national guidelines. Stress ulcer prophylaxis was not routinely used. Selective digestive decontamination was not used.

### Data collection

Data related to VAP and VAT episodes were prospectively collected. Data regarding mechanical ventilation (PEEP and FiO2) were automatically imported every hour in the patient management software ICIP^®^ (Philips Healthcare). Data from 2-h time slots were then retrospectively examined to determine the episodes of VAE. Other data such as body temperature, leukocytosis, antibiotic use, patient characteristics, aetiology of VAE episodes, duration of mechanical ventilation and hospitalization, and mortality were collected retrospectively from patients’ computerized medical records.

### Statistical analysis

The incidence rate and Cohen’s kappa coefficients were calculated on all episodes of VAE, VAP and VAT. The concordance between the diagnosis for VAC, IVAC, pVAP and that of VAP and VAT was determined by Cohen’s kappa statistic [10].

Only first episodes were taken to examine patients’ characteristics according to the occurrence of VAE, VAP and VAT. Qualitative variables were expressed in percentage. Because of non-normal distribution, quantitative variables were expressed in median, 25th and 75th percentiles. Chi squared test, or Fisher’s exact test; and Mann–Whitney U test, or Kruskal–Wallis test, were used to compare the qualitative and quantitative variables, respectively. The difference was considered significant when *p* < 0.05. When a significant difference existed between patients with VAP, VAT, and those with no VA-LRTI, comparisons between different groups were performed: VAP vs VAT, VAP vs no VA-LRTI and VAT vs no VA-LRTI.

Multivariate analyses, using forward multiple logistic regression models, were performed to determine the impact of VA-LRTI and VAE on mortality, adjusting for age, SAPS II and immunosuppression.

## Results

**Fig. 1:**
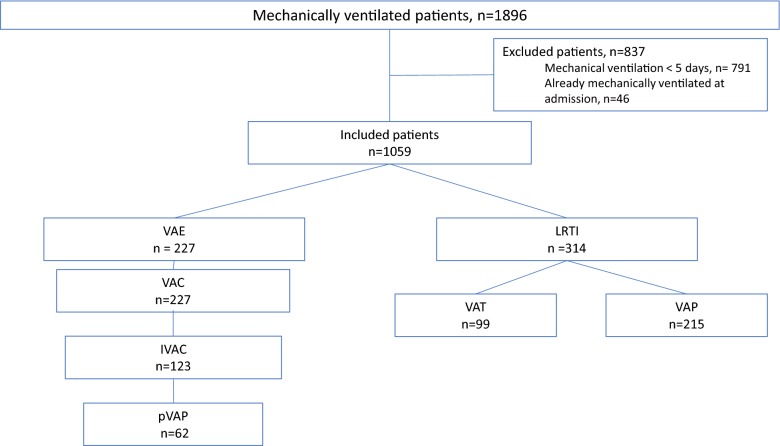
Flowchart. Data are number of patients. p, probable; IVAC, infectious ventilator-associated condition; VAC, ventilator-associated condition; VAP, ventilator-associated pneumonia; VAT, ventilator-associated tracheobronchitis

Out of the 1896 patients who received invasive mechanical ventilation, 837 (44%) were excluded (Fig. 1). Overall 1059 patients were included and received 15,029 days of invasive mechanical ventilation.

### Incidence of VAE and VA-LRTI

A total of 262 episodes of VAC (17.4 for 1000 ventilator-days), 268 VAP (17.8 for 1000 ventilator-days) and 127 VAT (8.5 for 1000 ventilator-days) were diagnosed and used for concordance analysis.

At least one episode of VAC, IVAC, and pVAP was diagnosed in 227 (21.4%), 123 (11.6%), and 62 (5.9%) patients, respectively. At least one episode of VAT or VAP were diagnosed in 99 (9.3%) and 215 (20.3%) patients, respectively. Among patients with VAT, 12 (9.4%) patients developed a subsequent VAP.

### Correlation between VAE and VA-LRTI

**Fig. 2:**
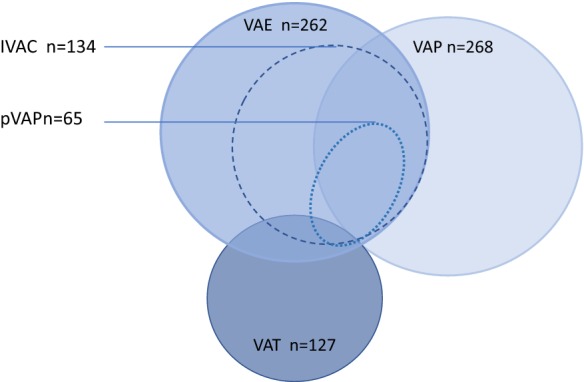
Consistency between VAE and VA–LRTI diagnoses. Data are number of VAE, VAP, or VAT. All episodes of VAE, VAP or VAT were taken into account. p, probable; IVAC, infectious ventilator-associated condition; VAC, ventilator-associated condition; VAE, ventilator-associated events; VAP, ventilator-associated pneumonia; VAT, ventilator-associated tracheobronchitis

A total of 6 VAT and 71 VAP were correlated with VAC (Fig. 2). Concordance (kappa statistic) between VAC and VAP, IVAC and VAP, pVAP and VAP were 0.03 (95% CI 0–0.11), 0.15 (95% CI 0.07–0.23), and 0.27 (95 CI 0.18–0.35), respectively (Table 1). Because of the small number of patients with VAE and VAT (*n* = 6), kappa statistic could not be calculated.

**Table 1:** Correlation between ventilator-associated events and ventilator-associated lower respiratory tract infections

	VAC, *n* = 262	IVAC, *n* = 134	pVAP, *n* = 65
VAP	71 (27.1)	58 (43.3)	56 (86.2)
No VAP	191 (72.9)	76 (56.7)	9 (13.8)

	K_VAP-VAC_ = 0.03 (0–0.11)	K_VAP-IVAC_ = 0.15 (0.07–0.23)	K_VAP-pVAP_ = 0.27 (0.18–0.35)
VAT	6 (2.3)	3 (2.2)	3 (4.6)
No VAT	256 (97.7)	131 (97.8)	62 (95.4)

Data are numbers (%)

The correlation between ventilator-associated events and VAT could not be calculated because of the small number of patients with VAC and VAT (*n* = 6)

IVAC, infection-related ventilator-associated condition; p, probable; VAC, ventilator-associated condition; VAP, ventilator-associated pneumonia; VAT, ventilator-associated tracheobronchitis

### Clinical significance of VAE

**Fig. 3:**
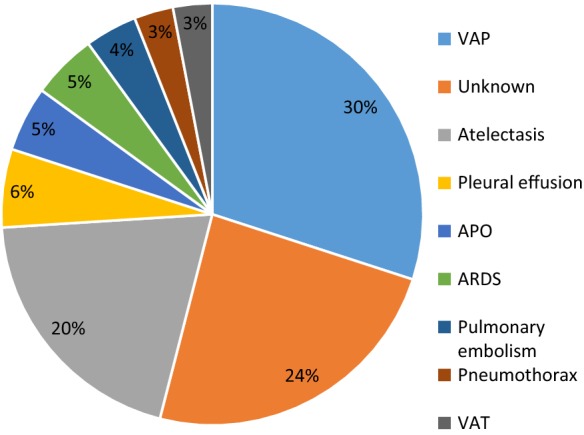
Clinical causes for VAE. APO, acute pulmonary oedema; ARDS, acute respiratory distress syndrome; VAP, ventilator-associated pneumonia; VAT, ventilator-associated tracheobronchitis

The most common causes for VAE were VAP (*n* = 79, 30%), and atelectasis (*n* = 53, 20%). 2.6% of VAE were possibly related to VAT, and no aetiology was found for (63, 24%) episodes (Fig. 3).

### Patient characteristics based on VA-LRTI

Characteristics of patients who presented VAT, VAP, or no VA-LRTI are presented in Table 2. A significant difference was found between the three groups regarding male gender, age, neurologic failure at ICU admission, and immunosuppression. Median duration of mechanical ventilation before VAT, and VAP occurrence was 9 (5, 13), and 7 (4, 13) days, respectively.

**Table 2:** Patient characteristics at ICU admission, based on the presence of ventilator-associated lower respiratory tract infections

	VAP, *n* = 215	VAT, *n* = 99	No VA-LRTI, *n* = 745	*p*
Male gender	156 (72.5)	66 (66.6)	473 (63.5)	0.001^#^
Age, years	57 (45–67)	59 (47–69)	61 (49–69.8)	0.041^#^
SAPS II	59 (48–73)	55 (45–68)	58.5 (46–71)	0.126
SOFA	10 (7–12)	9 (6–11)	9 (6–12)	0.091
Reason for admission				
Medical vs surgical	181 (84.2)	80 (80.8)	622 (83.5)	0.742
Septic shock	54 (25.1)	17 (17.2)	194 (26)	0.158
Acute respiratory failure	80 (37.2)	35 (35.4)	260 (34,9)	0.830
Neurologic failure	53 (24.7)	28 (28.3)	129 (17.3)	0.005^#^*
Soft tissue infection	15 (7)	12 (12.1)	79 (10.6)	0.224
Cardiac arrest	22 (10.2)	10 (10.1)	88 (11.8)	0.741
Poisoning	8 (3.7)	2 (2)	37 (5)	0.345
Other	35 (16.3)	12 (12.1)	115 (15.4)	0.624
Charlson comorbidity index	3 (1–5)	3 (1–5)	4 (2–6)	0.660
Diabetes	43 (20)	20 (20.2)	177 (23.8)	0.417
Chronic renal failure	17 (7.9)	13 (13.1)	75 (10.1)	0.343
Chronic heart failure	37 (17.2)	21 (21.2)	132 (17.7)	0.665
Chronic respiratory failure	15 (15.9)	7 (10.9)	57 (14.7)	0.885
Cirrhosis	18 (8.4)	8 (8)	67 (7.7)	0.918
Immunosuppression	59 (27.4)	17 (17.2)	215 (28.9)	0.049*^¤^
Obesity				
BMI > 30	50 (23.3)	26 (26.3)	141 (18.9)	0.128
BMI > 35	27 (12.6)	11 (11.1)	78 (10.5)	0.320

Results are numbers (%), or median (interquartile range)

BMI: body mass index; SAPS, simplified acute physiology score; SOFA, sepsis-related organ failure assessment; VA-LRTI, ventilator-associated low respiratory tract infection; VAP, ventilator-associated pneumonia; VAT, ventilator-associated tracheobronchitis

* Significant difference between VAT and no VA-LRTI groups

^#^Significant difference between VAP and no VA-LRTI groups

^¤^Significant difference between VAP and VAT groups

### Patient characteristics based on VAE

The characteristics of patients with VAC, IVAC, or pVAP, and those without these conditions are presented in Table 3. In VAC patients, as compared with those with no VAC, male gender, SOFA at ICU admission, and BMI > 30 were significantly higher. In IVAC patients, as compared with those with no IVAC, SOFA score at ICU admission, and BMI > 30 were significantly higher. In pVAP patients, as compared with those with no pVAP, age, medical category, and Charlson comorbidity index were significantly lower. Median duration of mechanical ventilation before VAC, and iVAC, and pVAP occurrence was 5 (3, 9), 5 (3, 9), and 5 (4, 10) days, respectively.

**Table 3:** Patient characteristics at ICU admission, based on the presence of ventilator-associated events

	VAC, *n* = 227	No VAC, *n* = 832	*p*	IVAC, *n* = 123	No IVAC, *n* = 936	*p*	pVAP, *n* = 62	No pVAP, *n* = 997	*p*
Male gender	164 (72.2)	531 (63.8)	0.020	88 (71.5)	607 (64,9)	0.150	44 (71)	651 (65.3)	0.372
Age, years	59 (47–67)	61 (47–70)	0.227	58 (46–67)	61 (47–69.7)	0.121	55 (45–66)	61 (47–70)	0.006
SAPS II	56 (46–74)	59 (46–70)	0.804	58 (44–72)	58 (46–70)	0.353	60 (47–73)	58 (46–71)	0.192
SOFA	9 (7–12)	9 (6–11)	0.041	9 (6–12)	10 (6.25–11)	0.009	9 (7–13)	9 (7–12)	0.392
Reason for admission									
Medical vs surgical	189 (83.3)	693 (83.3)	0.933	94 (76.4)	788 (84.2)	0.026	46 (74.2)	836 (83,9)	0.043
Septic shock	65 (28.6)	200 (24)	0.162	32 (26)	233 (24.9)	0.797	14 (22.6)	251 (25.1)	0.641
Acute respiratory failure	93 (41)	282 (33,9)	0.051	43 (35)	332 (35.5)	0.898	18 (29)	357 (35.8)	0.274
Neurologic failure	39 (17.2)	171 (20.6)	0.252	21 (17.1)	189 (20.2)	0.409	11 (17.7)	199 (20)	0.665
Soft tissue infection	23 (10.1)	83 (10)	0.953	16 (13)	90 (9.6)	0.242	8 (12.9)	98 (9.8)	0.437
Cardiac arrest	21 (9.3)	98 (11.8)	0278	13 (10.1)	106 (11.3)	0.794	9 (14.5)	110 (11.1)	0.405
Poisoning	7 (3.1)	40 (4.8)	0.261	4 (3.3)	43 (4.6)	0.343	2 (3.2)	45 (4.5)	0.631
Other	44 (19.4)	118 (14.2)	0.056	28 (22.8)	134 (14.3)	0.015	12 (19.4)	150 (15.1)	0.364
Charlson comorbidity index	3 (2–5)	4 (1–5)	0.440	3 (1–5)	4 (2–5)	0.639	3 (2–5)	4 (1–5)	0.024
Diabetes	58 (25.6)	182 (21.9)	0.248	33 (26.8)	207 (22.2)	0.246	13 (21)	227 (22.8)	0.736
Chronic renal failure	17 (7.5)	88 (10.6)	0.899	9 (7.3)	96 (10.3)	0.302	3 (4.8)	102 (10.3)	0.167
Chronic heart failure	40 (17.6)	150 (18.1)	0.875	22 (17.9)	168 (18)	0.978	10 (16.1)	180 (18.1)	0.696
Chronic respiratory failure	36 (15.9)	120 (14.5)	0.598	19 (15.4)	137 (14.7)	0.819	10 (16.1)	146 (14.7)	0.754
Cirrhosis	23 (10.1)	70 (8.4)	0.436	13 (10.6)	80 (8.6)	0.470	6 (9.7)	87 (8.7)	0.809
Immunosuppression	55 (24.2)	236 (28.4)	0.209	33 (26.8)	258 (27.6)	0.853	17 (27.4)	274 (27.5)	0.984
Obesity									
BMI > 30	64 (28.2)	153 (18.4)	0.001	35 (28.5)	182 (19.5)	0.021	8 (29)	199 (20)	0.088
BMI > 35	30 (13.2)	86 (10.3)	0.223	18 (14.6)	98 (10.5)	0.167	10 (16.1)	106 (10.6)	0.181

Data are numbers (%), or median (interquartile range)

BMI, body mass index; SAPS: simplified acute physiology score; SOFA, sepsis-related organ failure assessment

### Outcomes based on VA-LRTI

ICU mortality, duration of mechanical ventilation and length of stay were significantly different between patients with VAP, VAT, or no VA-LRTI (Table 4). In patients with VAP, as compared with those with no VA-LRTI, duration of mechanical ventilation, and length of stay were significantly higher. In patients with VAP, as compared with those with VAT, ICU mortality was significantly higher. In patients with VAT, as compared with those with no VA-LRTI, duration of mechanical ventilation and length of stay were higher, and ICU mortality was significantly lower. In multivariate analysis, the occurrence of VA-LRTI was not associated with mortality (Table 6).

**Table 4:** Outcomes of study patients based on the presence of ventilator-associated lower respiratory tract infections

	VAP, *n* = 215	VAT, *n* = 99	No VA-LRTI, *n* = 745	*p*
ICU mortality	82 (38.1)	20 (20.2)	313 (42)	< 0.001*^¤^
Duration of mechanical ventilation, days	18 (11–31)	18 (11–24)	8 (6–13)	< 0.001#*
Mechanical ventilation-free days	1 (0–13)	8 (0–15)	11 (0–20)	< 0.001^#^*^¤^
Length of ICU stay, days	26 (15–42)	25 (17–37)	12 (8–18)	< 0.001^#^*
Length of hospital stay, days	31 (18–60)	36 (24–56.3)	17 (10–30)	< 0.001^#^*

Data are numbers (%), or median (interquartile range)

d, days; VAP, ventilator-associated pneumonia; VAT, ventilator-associated tracheobronchitis

* Significant difference between VAT and no VA-LRTI groups

^#^Significant difference between VAP and no VA-LRTI groups

^¤^Significant difference between VAP and VAT groups

### Outcomes based on VAE

Although duration of mechanical ventilation and length of stay were significantly higher in patients with VAC, IVAC, or pVAC, as compared with those without these conditions, no significant difference in mortality was found between these different groups (Table 5). In multivariate analysis, the occurrence of VAE, or of VA-LRTI was not associated with mortality (Table 6).

**Table 5:** Outcomes of study patients based on the presence of ventilator-associated events

	VAC,* n* = 227	No VAC,* n *= 832	*p*	IVAC, *n* = 123	No IVAC, *n* = 936	*p*	pVAP, *n* = 62	No pVAP, *n* = 995	*p*
ICU mortality	98 (43.2)	316 (38)	0.163	23 (45.1)	340 (36.3)	0.180	26 (41.9)	388 (39)	0.547
Duration of mechanical ventilation, days	15 (10–27)	9 (6–15)	< 0.001	16 (12.5–29)	9 (6–16)	< 0.001	18 (15–31)	9 (6–16)	< 0.001
Mechanical ventilation-free days	1 (0–11)	9 (0–16)	< 0.001	1 (0–10)	8 (0–15)	< 0.001	5 (0–14)	8 (0–15)	0.002
Length of ICU stay, days	19 (13–32)	14 (9–23)	< 0.001	20 (16–31.5)	14 (9–24)	< 0.001	23 (19–34)	14 (9–24)	< 0.001
Length of hospital stay, days	27 (17–51)	19 (11–35)	< 0.001	27 (18.5–52)	20 (11–35.5)	< 0.001	28 (19.5–53)	20 (11–36)	< 0.001

Data are numbers (%), or median (interquartile range)

d, day; IVAC, infection-related ventilator-associated condition; p, probable; VAC, ventilator-associated condition

**Table 6:** Risk factor for ICU mortality by multivariate analysis

	OR (95% CI)	*p* value
Model 1		
Immunosuppression	2.1 (1.6–2.8)	< 0.001
Age, years	1.02 (1.01–1.03)	< 0.001
SAPS II	1.02 (1.01–1.04)	< 0.001
VA-LRTI		0.128
VAT	–	–
VAP	–	–
No infection	–	–
Model 2		
Immunosuppression	2.1 (1.6–2.8)	< 0.001
Age, years	1.02 (1.01–1.03)	< 0.001
SAPS II	1.02 (1.01–1.04)	< 0.001
VAE	–	0.149

Hosmer and Lemeshow goodness-of-fit test *p* = 0.39, and *p* = 0.46 for models 1, and 2, respectively

SAPS, simplified acute physiology score; VA-LRTI, ventilator-associated lower respiratory tract infections; VAE, ventilator-associated events

## Discussion

Our results suggest that VAE are moderately correlated to VAP, and not correlated to VAT. VAE and VA-LRTI are all associated with increased duration of mechanical ventilation and length of hospital and ICU stay. VA-LRTI and VAE were not independently associated with mortality.

The strengths of our study include the large number of included patients (n = 1059) and ventilator days (n = 15,029), and the prospective evaluation of all VA-LRTI, including VAT. Previous studies reported similar findings regarding the correlation between VAE and VAP [6, 11, 12]. However, few studies prospectively evaluated the incidence of VAT in patients with VAE [11, 13]. Among the 262 diagnosed episodes of VAE, only 6 (2%) were possibly related to VAT. Thus, the correlation between VAE, and VAT could not be calculated. In a retrospective analysis of prospectively collected data, Bouadma et al. [11] identified aetiologies of each episode and found only 1% of VAC caused by tracheobronchitis.

Worsening of ventilatory parameters is not a mandatory criterion for VA-LRTI definition, and only the Clinical Pulmonary Infection Score (CPIS) includes the alteration of the PaO_2_/FiO_2_. Our study and previous studies [5, 11] reported that an important percentage of VAE episodes were possibly related to VAP, and not to VAT. This clearly suggests that using alteration of oxygenation, i.e. PaO_2_/FiO_2_, could be helpful in differentiating VAP from VAT. Differentiating these two infections could be a difficult task, as the accuracy of chest X-ray in diagnosing new infiltrates is low [14–16]. However, it is still important to differentiate them, as antibiotic treatment is not recommended for VAT and inappropriate use of antimicrobials is a risk factor for subsequent emergence of multidrug-resistant bacteria [17, 18]. Previous large observational studies and two small randomized controlled trials suggested beneficial effects of systemic and inhaled antibiotics. However, several limitations preclude definite conclusions on the interest of antimicrobials in patients with VAT, and further large multicentre randomized controlled trials are required.

The incidence of VAE in our study is in line with previous findings. However, the incidence of VAP (21%) is somehow higher than that reported by recent studies [19]. This could be explained by the fact that only patients receiving mechanical ventilation for > 4 days were included in our study.

VAE, and VA-LRTI were associated with significantly longer duration of mechanical ventilation and hospital and ICU stay. However, VAE and VA-LRTI were not independently associated with mortality. In contrast, two previous studies reported that VAE were associated with significantly higher mortality rates [11, 12].

VAE diagnosis algorithm is based on objective criteria and easy to use in routine in mechanically ventilated patients. However, the clinical relevance of VAE is not clear. First, our study and previous ones clearly showed that applying a VAE algorithm surveillance is not accurate in detecting VA-LRTI. Second, the impact of a ventilator bundle on VAE incidence is unknown. Few studies have focused on the preventability of VAE, but it seems that bundles applied for VAP prevention are not completely effective for VAE prevention [20–22]. Our results show that many VAE were non-infectious events as atelectasis, pleural effusion or acute pulmonary embolism. This might explain why ventilator bundles are not effective in preventing VAE, and should not be used to assess the quality of care in mechanically ventilated patients.

VAE algorithm failed in identifying most of VAT and 73% of VAP episodes and should not be used to start an empirical treatment of VA-LRTI. Furthermore, the retrospective nature of VAE does not allow its use at bedside. In fact, to meet IVAC criteria, patients must have 2 days of stable oxygenation parameter and 2 days of worsening ventilatory settings after which a new antibiotic must be prescribed for at least 4 days.

Our study has some limitations. First, it was performed in a single center, and its results may not be generalized to all ICU patients. Second, it was retrospective. However, all VA-LRTI were prospectively identified. Third, no data were collected on duration of antibiotic treatment before VA-LRTI, and VAE, neither on appropriateness of antibiotic treatment in patients with VA-LRTI. Fourth, the definition of VAT and VAP was based on chest X-ray that was interpreted by physicians in charge and no blind interpretation was performed. The prolonged duration of mechanical ventilation reported in patients with VA-LRTI, or VAE, as compared to those with no infection, or no VAE could not be attributed to these events as no adjustment was performed.

## Conclusion

VAE and VA-LRTI are common in mechanically ventilated critically ill patients, and have a significant impact on duration of mechanical ventilation and length of stay. VAE are moderately correlated to VAP, and not correlated to VAT. Our results suggest that VAE should not be used as a marker of quality of care or to start empirical antibiotic treatment.

## Supplementary information

**Additional file 1.** Online supplementary material.

## Data Availability

All data are provided in the manuscript.

## Abbreviations

CI: confidence interval
CPIS: clinical pulmonary infection score
OR: odds ratio
RGV: residual gastric volume
VAP: ventilator-associated pneumonia
VAT: ventilator-associated tracheobronchitis
