# Supplementary material for: Accuracy of ventilator-associated events for the diagnosis of ventilator-associated lower respiratory tract infections
Source: Ann Intensive Care. 2020 Jan 13;10:6. doi: 10.1186/s13613-020-0624-6 (PMC6957592; doi:10.1186/s13613-020-0624-6)
Supplement: Supplementary file 1 — Additional file 1. Online supplementary material. [file 13613_2020_624_MOESM1_ESM.docx]

**Additional file 1**

**Accuracy of ventilator-associated events for the diagnosis of ventilator-associated lower respiratory tract infections**

Olivier POULY^1,2^, Sylvain LECAILTEL^3^, Sophie SIX^1^, Sébastien PREAU^1^, Frédéric WALLET^4^,

Saad NSEIR^1,2^, Anahita ROUZE^1^

^1^Critical Care Center, CHU Lille, F-59000 Lille, France

^2^Lille University, Medicine Faculty, F-59000 Lille, France

^3^Intensive Care Unit, CH de Boulogne, Boulogne, France

^4^Centre de Biologie et de Pathologie, CHU Lille, F-59000 Lille, France

**FIGURE LEGENDS**

Figure S1: Diagnostic criteria for VAE, according to CDC definition.

IVAC, infection related ventilator-associated condition; pVAP, probable ventilator-associated pneumonia; VAC, ventilator-associated condition

Figure S2: Criteria used to consider VA-LRTI and VAE episode as concomitant.

D, day; PEEP, positive end expiratory pressure; VAE, ventilator-associated events; VA-LRTI, ventilator-associated low respiratory tract infection
